# Measurements of the local evoked potential from the cochlear nucleus in patients with an auditory brainstem implant and its implication to auditory perception and audio processor programming

**DOI:** 10.1371/journal.pone.0249535

**Published:** 2021-04-01

**Authors:** Lutz Gärtner, Thomas Lenarz, Andreas Büchner

**Affiliations:** Department of Otolaryngology, Hannover Medical School, Hannover, Germany; University of California, Los Angeles, UNITED STATES

## Abstract

The measurement of the electrically evoked compound action potential (ECAP) in cochlear implant (CI) patients is widely used to provide evidence of a functioning electrode-nerve interface, to confirm proper location of the electrode array and to program the sound processor. In patients with an auditory brainstem implant (ABI), a likewise versatile measurement would be desirable. The ECAP measurement paradigm “Alternating Polarity” was utilized to record responses via the implanted ABI electrode array placed on the cochlear nucleus. Emphasizing on the different location of stimulation and recording, these responses are called local evoked potentials (LEP). LEP measurements were conducted during the clinical routine in 16 ABI patients (12 children and 4 adults), corresponding to 191 electrode contacts. A retrospective analysis of these data revealed, that LEP responses were observed in 64.9% of all measured electrode contacts. LEP responses predicted auditory perception with a sensitivity of 90.5%. False-positive rate was 33.7%. Objective LEP thresholds were highly significantly (p < 0.001) correlated both to behavioral thresholds (Pearson’s r = 0.697) and behavioral most comfortable levels (r = 0.840). Therefore, LEP measurements have the potential to support fitting in ABI patients.

## Introduction

Originally, an auditory brainstem implant (ABI) was used to restore hearing after tumor resection in patients with neurofibromatosis type 2 (NF2) [1]. In the meantime, patients with congenital malformation of the inner ear or with cochlear nerve aplasia, in which case a cochlear implant (CI) would not provide sufficient hearing, have been benefitting from an ABI, too. By now, over one thousand of such devices have been implanted worldwide [2]. In order to cope with the age-related development of the auditory cortex, early implantation of deaf children is recommended [3]. However, fitting the sound processor can be challenging, especially in the case of very young children or other patients, who cannot give sufficient feedback regarding their hearing perception. In CI users, the electrically evoked compound action potential (ECAP) is used as an objective measure to check the electrode-nerve interface as well as to predict, to a certain amount, the most important behavioral fitting parameters [4]. These are most comfortable level (MCL), i.e. a loudness level that is well tolerable for the implant user, and threshold level (THR). CI manufactures have implemented a method into their clinical software to record ECAPs via the patient’s sound processor connected to a computer. In comparison to the measurement of the electrically evoked auditory brainstem response (EABR), no additional equipment is necessary and the patient does not need sedation. Using postoperative ECAP measurements, also allows for proving the proper location of the CI electrode without the burden of radiation [5]. Such measurements should also be possible for ABI users via their ABI electrode array. However, only few publications on this topic have been available so far (e.g. [6,7]), and the benefit of measuring electrophysiological responses via the ABI electrode array has been contested [8,9]. One reason for the lack of success could be an unsuitable artifact reduction paradigm. To measure ECAP responses in CI, an artifact reduction paradigm like “Forward Masking” (FM) or “Alternating Polarity” (AP) [10] is necessary to separate the small response from the large stimulus artifact. While using FM in measurements with ABI patients, Azadpour et al. [11] observed facilitation where masking was expected. From this, we assume that FM may not work as artifact reduction paradigm in ABI patients and that AP might be the better way to record responses.

**Fig 1:**
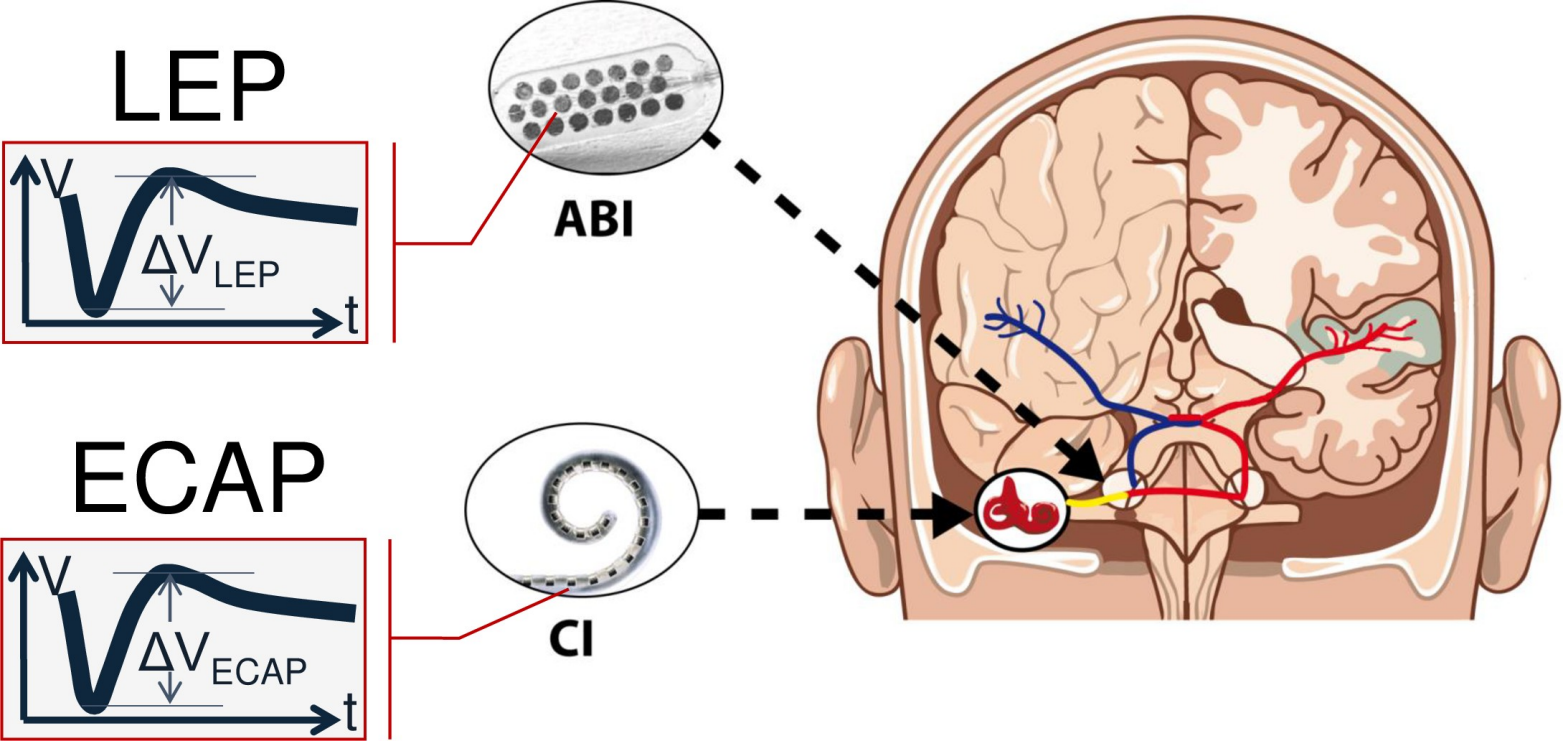
Schematic diagram of ECAP measurements in CI patients and LEP measurements in ABI patients. For illustration purposes, an arbitrary response (voltage V over time t) is shown. With respect to the different locations of stimulation and recording, responses from an ABI are called “Local Evoked Potentials” (LEP).

In CI users, ECAP measurements are related to responses, which were recorded with an electrode array placed inside the cochlea, usually inside the scala tympani. In contrast, the ABI electrode array is placed onto the cochlear nucleus. Therefore, different neuronal populations will respond to an electric stimulus. To emphasize on these differences, we call the kind of response “Local Evoked Potential” (LEP) (Fig 1).

To check the electrode-neuron interface, ECAPs are measured routinely in our CI users, both intraoperatively as well as postoperatively. When over time ECAP responses become absent or ECAP thresholds are going to deviate considerably, an electrode migration can be suspected [5]. By attempting to find a method to verify proper electrode location without radiation exposure, LEP measurements have now become clinical routine at our university.

In this retrospective study, we analyzed to which extend our measurements would predict auditory perception and fitting parameters.

## Methods

### Ethics statement

This publication contains data collected during the clinical routine and is approved by the ethics board of the Hannover Medical School (No. 1897–2013). The Ethics Board approval covers evaluation of all data acquired during clinical routine regardless of age. The ethics committee waived the requirements for informed consent.

### Subjects

17 patients were implanted with a MED-EL ABI at our clinic until end of 2018. Demographical data are shown in Table 1. All children have aplasia of the VIII^th^ nerve. Four of them (A-06, A-07, A-08, A-12) were first implanted with a CI without showing auditory perception. A-02 underwent revision surgery due to electrode migration at the age of 3.18 years. A-03 was re-implanted at the age of 3.39 years, with revision surgery due to electrode migration at the age of 4.38 years. Patients with NF2 are A-13, A-14, and A-16. A-16 passed away before the initial fitting. A-15 became deaf after meningitis and was bilaterally implanted alio loco with a CI. Since no auditory perception could be elicited, an ABI was implanted 9 months later. A-17 has otosclerosis. He was implanted with a CI at the age of 40.8 years. Over time, stimulus charge had to be increased to maintain sufficient auditory perception. However, due to increasing facial nerve stimulation, half the channels in the medial to basal region had to be deactivated and after 20 years of CI usage, he was provided with an ABI.

**Table 1:** Subject demographics.

ID	Gender	Age at implantation [years]	Implanted side	Implant type	Period of implant use at last CAP value estimation [years]	Last CAP value
A-01	m	1.84	L	C	4.79	4
A-02	f	2.69	R	S	1.44	0
A-03	m	2.93	L	S	1.35	0
A-04	m	2.99	R	C	4.57	1
A-05	m	3.13	R	S	0.01	0
A-06	m	3.25	L	S	3.36	4
A-07	f	3.34	R	S	0.86	4
A-08	m	4.04	R	S	0.30	1
A-09	f	4.05	R	S	0.38	2
A-10	f	4.11	L	S	0.84	4
A-11	m	4.37	R	S	3.19	4
A-12	m	6.59	R	C	4.83	0
A-13	m	20.85	R	S	0.01	4
A-14	m	26.57	L	S	0.36	0
A-15	m	47.25	R	P	9.18	4
A-16	m	50.02	R	S	n/a	n/a
A-17	m	60.82	R	C	4.51	4

The ABI implant type was either a MED-EL Concerto ABI (C), a MED-EL Pulsar ABI (P) or a MED-EL Synchrony ABI (S). Speech recognition is described by categories of auditory performance (CAP).

The benefit of speech recognition can be described by the category of auditory performance, CAP [12]. CAP 0 characterizes lacking awareness of environmental sound or voices. With CAP 1, some reaction to different environmental sounds can be established. The classification CAP 2 describes response to speech sounds. If environmental sounds can be identified, CAP 3 will be rated. CAP 4 indicates discrimination of at least two speech sounds without lip-reading. Higher scores include cognitive abilities and indicate understanding of simple phrases up to conversation via telephone and in noisy environments. Like in CI patients, CAP is expected to increase with period of ABI use. The CAP achieved at the last follow-up visit, is shown in Table 1.

### Fitting

The fitting of the ABI audio processor was carried out behaviorally using the clinical software MAESTRO (MED-EL, Austria). In all cases, the initial fitting was conducted 4 to 8 weeks after implantation. HDCIS (high definition continuous interleaved sampling) was used as speech coding strategy. The stimulus charge on each electrode contact was increased slowly up to a level where either a clear auditory perception was elicited or side effects occurred or the highest possible charge was reached. The maximum pulse duration (PD) available for stimulation in MED-EL ABI devices is limited to approximately 200 μs. Due to compliance limits, the respective maximum charge depends on the impedance of the electrode contact, but will not exceed 240 nC per phase. To determine behavioral levels (THR and MCL), pulse trains of a pulse rate equaling the stimulation rate of the fitting map, was used. This rate was typically set in the range of a few hundred pulses per second (pps). In many cases, higher rates were not achievable due to the need for high stimulus charge levels (see S1 Table for details). In children, the initial fitting took usually 3–5 days. Follow-up visits were scheduled every 6 weeks. Assuming a slow development of auditory perception, an electrode contact remained activated for the first months, despite a lack of behavioral reaction indicating auditory perception. However, whenever typical side effects were observed, i.e. facial nerve stimulation or coughing, the channel was deactivated immediately.

In adults, initial fitting took 5 days and was repeated after 3 and 6 months. Thereafter, follow-up visits are organized annually. Only electrodes eliciting auditory perception have been activated. In addition to the estimation of behavioral parameters THR and MCL, a pitch ranking procedure accompanied each fitting.

Results of LEP measurements were never used for fitting.

### Local evoked potential (LEP)

LEP was measured utilizing the ART (auditory nerve response telemetry) task within the clinical software MAESTRO. The patient’s audio processor with the measurement coil placed over the implant, was connected to a personal computer via the MAX interface box.

Comparable to ECAP measurements in CI patients, biphasic, charge-balanced stimuli were delivered with increasing current to one electrode contact of the ABI array. The response was recorded from an adjacent electrode contact. The measurement window captured responses up to 1.7 ms after stimulus onset. The stimulation rate of the presented stimuli was at approximately 76 pps.

LEP measurements were carried out on all electrode contacts, regardless whether they were activated or deactivated in the fitting map. With occurrence of side effects or unpleasant feelings or overly loud sensations, the measurement was stopped immediately.

The recording electrode was either determined by the default values of the clinical software or differently chosen by the audiologist. Different recording electrodes were tried, e.g. if a response appeared to be very noisy or the amplifier inside the implant was clipping.

To reduce the stimulus artifact, AP is used within the ART task per default. Two stimuli are applied consecutively, one with the anodic leading phase and another with the cathodic leading phase. By averaging the responses of these two pulses, the artifact can be reduced [10]. In addition, the zero amplitude template subtraction algorithm, implemented in the ART task, was applied to all recordings to compensate for the amplifier artifact. Thereby, also a linear drift artifact will be removed automatically through rectification. According to the MAESTRO software user manual, “a straight line is adapted to and subtracted from each result curve in the range between 655 and 1402 μs so that each result curve ends with a 0 cu amplitude”.

Results from measurements taken by the AutoART task, benefitting from the fine-grain recording of the response [13], were additionally used in subject A-01. Only in this case, a different stimulation rate of 60 pps was used.

Responses sometimes were characterized by a negative (N) and a positive (P) peak. The LEP amplitude (ΔV_LEP_) was calculated based on their difference in voltage. In case no clear peaks were visible, the difference between minimum and maximum was used. An audiologist with experience in ECAP measurements for more than 15 years, estimated the objective threshold THRLEP. However, no standards regarding the assessment of LEP recordings have been established so far.

In MED-EL ABI implant devices, current units (cu) and charge units (qu) are approximately equal to SI (abbreviated from the French “Système international d’unités”, the “International System of Units”) units: 1 cu ≈ 1 μA, 1 qu ≈ 1 nC. Maximum stimulus charge of LEP measurement is given by maximum stimulus current (1200 cu) multiplied by maximum PD (100 μs), leading to 120 qu. In contrast to fitting procedure, where a programming map with PD up to 200 μs is possible, LEP measurements are limited to 100 μs. As mentioned previously, maximum stimulus charge may be further reduced by compliance limits.

LEP measurements were conducted during clinical routine, not within a prospective study. For that reason, no study protocol was used for systematically changing parameters like pulse duration, recording electrode, maximum stimulus current, and number of averages. The extent of measurements in each patient was variable and depended on his or her patience and the number of follow-up visits. Even in cases where no side effects occurred, individual LEP measurements were not necessarily performed at maximum stimulus charge possible. For our analysis, only the most recent measurements allowing for LEP-THR estimation have been used.

All data are included in S1 Table.

## Results

### LEP responses

In 16 patients (all but A-16), LEP have been measured on 191 different electrode contacts in total. One electrode contact (electrode E09 in subject A-01) had to be excluded due to high impedance.

**Fig 2:**
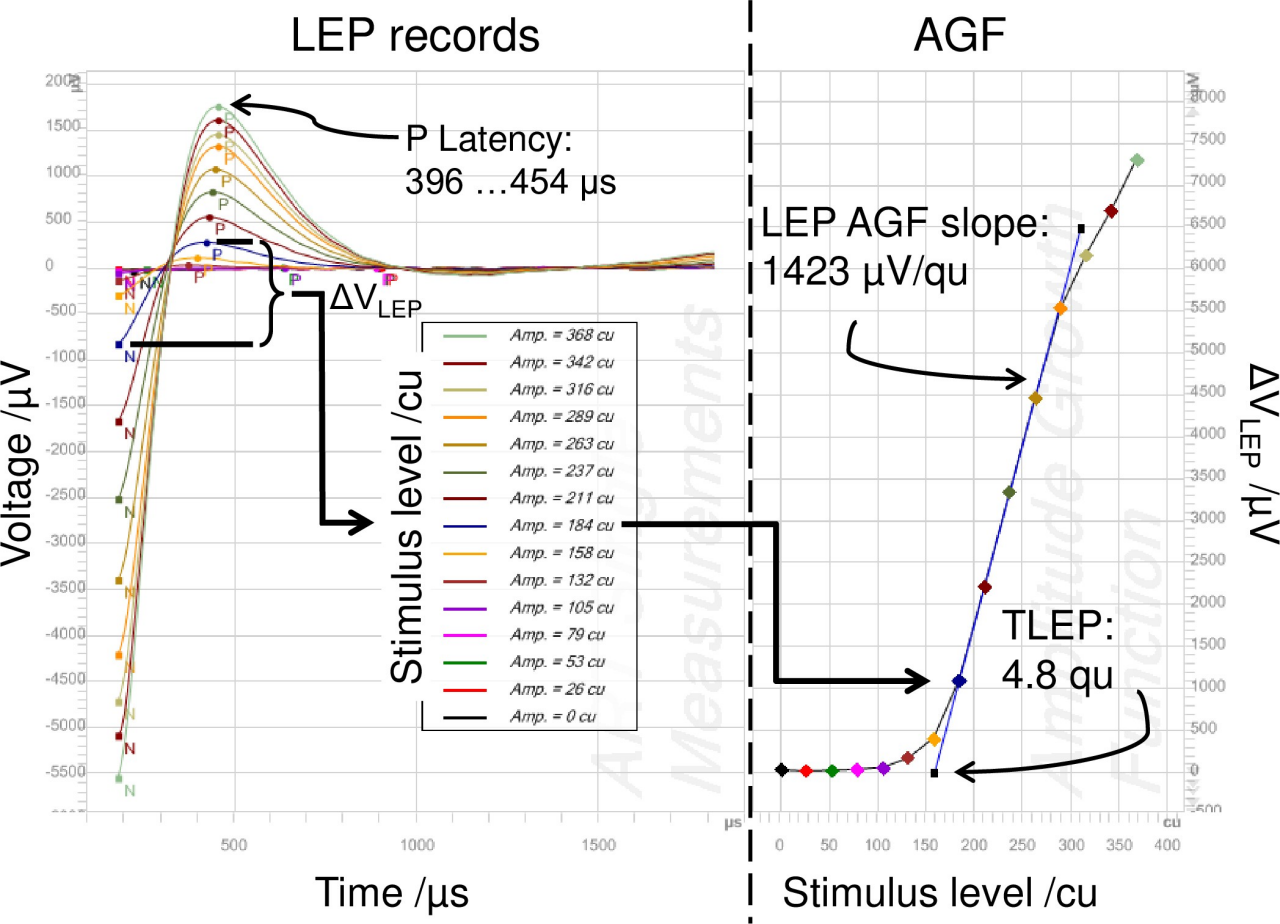
Example of a LEP measurement with clear responses. P peaks are well pronounced and the AGF appears unambiguous and steep with a broad linear region. Subject A-10, PD = 30 μs, stimulating electrode E05, recording electrode E04. TLEP = 159 cu corresponding to 4.8 qu, LEP slope = 42.68 μV/cu corresponding to 1422.83 μV/qu. Latencies of P peaks are between 396 and 454 μs after stimulus onset. See S1 Fig for raw responses.

Fig 2 shows an example with clear responses. On the left side, LEP records at different stimulus levels are put on top of each other. The x-axis shows time after stimulus onset. As an example, the LEP amplitude ΔV_LEP_ is highlighted for the stimulus level of 184 cu. On the right side, the amplitude growth function (AGF) derived from these records is shown. The slope was estimated from the linear region of the AGF. The objective LEP threshold (TLEP) was determined as the intersection point of this linear region with the x-axis. At high stimulus levels, a saturation effect could be observed. Besides shorter latencies of the P peaks, truncated N peaks, and much higher voltage, these LEP responses look similar to ECAP responses.

If a response has been observed, it in almost all cases showed the same pattern, i.e. a truncated N peak and a pronounced P peak. Only two recordings, those on contacts E02 and E05 of subject A-17, revealed a reversed polarity. Here, a falling edge at low latency and a negative peak at a latency of about 500 to 700 μs could be observed.

After examination of all available LEP recordings, they were classified whether a response occurred or not, based on the experience of assessing ECAP responses in CI users. From this analysis, we derived preliminary rules for rating recordings as LEP response.

**Fig 3:**
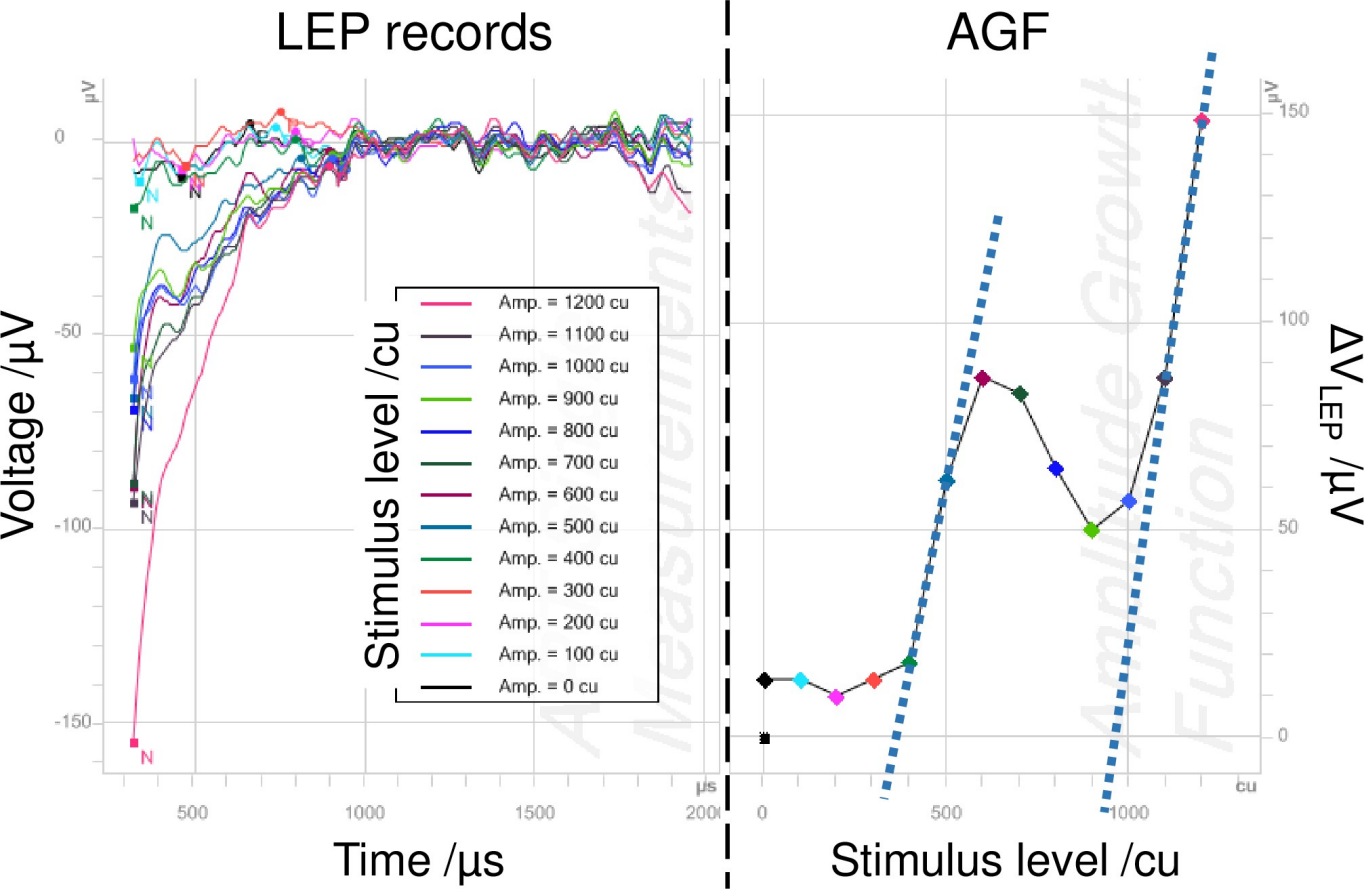
Example of a LEP measurement, which was not considered to contain a response. The AGF is ambiguous. Two linear regions (dotted lines) would lead to different LEP thresholds. Subject A-02, PD = 100 μs, stimulating electrode E02, recording electrode E01. See S2 Fig for raw responses.

The AGF should be mostly monotonically increasing with a linear region where several—at least three—measurement points with LEP amplitudes greater than 100 μV being aligned. It is important that the regression line through this linear region is not running through the origin, otherwise, it would indicate amplified noise only. In case of doubt, the presence of a pronounced P peak in the recording would speak in favor of a response. In some cases, the slope of the AGF is very shallow. Since in our CI users, slopes appear on average between 20 and 60 μV/qu, we decided to classify slopes less than 10 μV/qu as shallow. Fig 3 shows a recording, which was not classified as response, despite of some amplitude growth. The AGF is ambiguous with two linear regions, which would lead to different TLEPs. No clear peaks are observable. Only one measurement point exceeds a LEP amplitude of 100 μV.

### Correlation between LEP response and auditory perception

LEP responses were found on 124 (64.9%) out of 191 electrode contacts. On 95 contacts with responses, auditory perception has been elicited, too. However, on 10 contacts with auditory perception, LEP response was lacking. Therefore, the LEP method shows a 90.5% sensitivity.

On 57 contacts without auditory perception, no LEP responses were detected, while on 29 contacts no auditory perception was sensed but LEP responses observed. The specificity was hence 66.3%.

**Fig 4:**
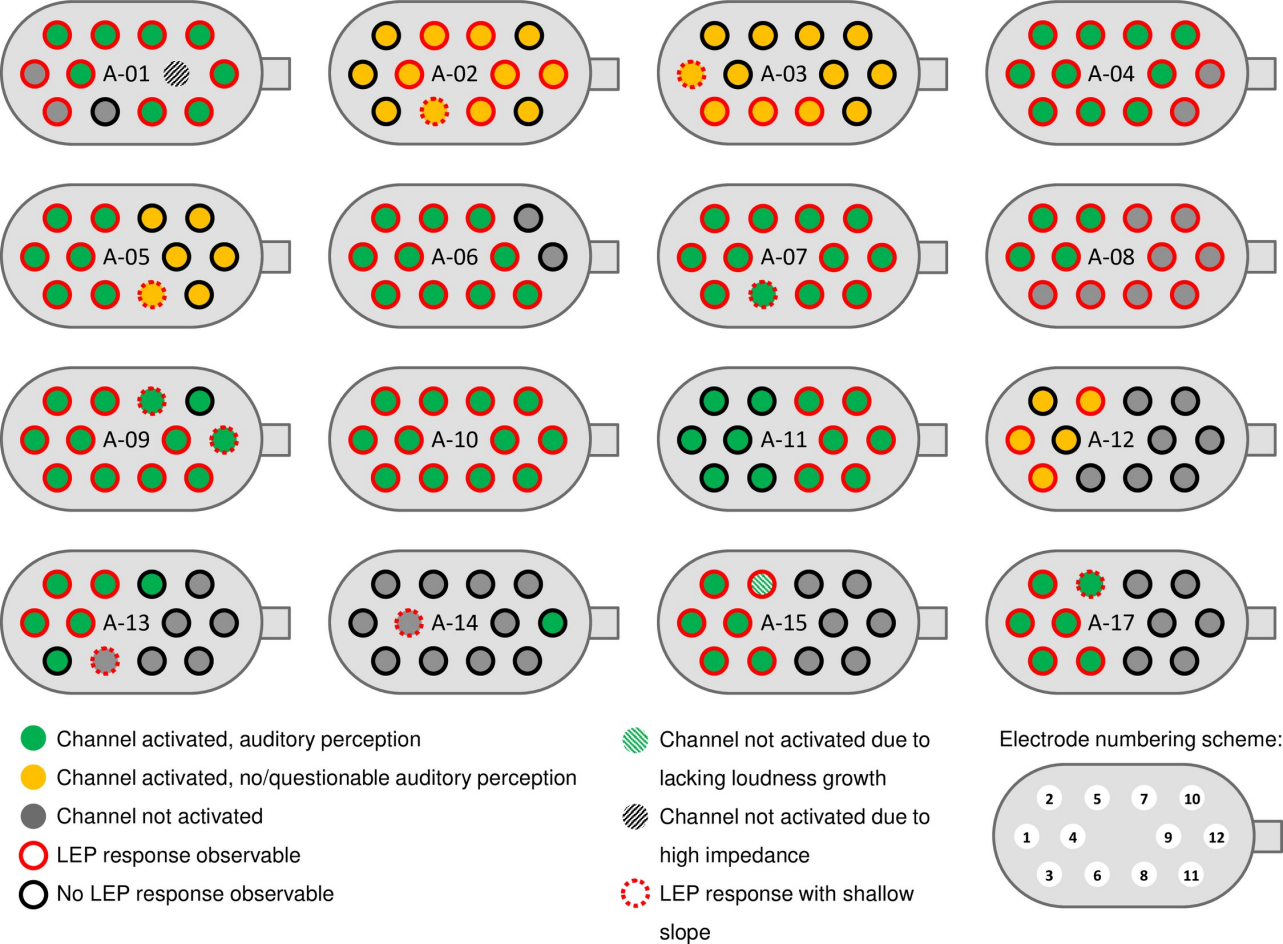
Electrode status for each patient. LEP measurements have been performed in 16 patients. The color indicates if an electrode contact was activated or deactivated and if LEP responses were found or not.

Fig 4 shows the status of each of the 12 electrode contacts of the ABI array in all 16 cases. On the bottom right, the order of the electrode contacts is shown with numbers ranging from E01 to E12. Green electrode contacts indicate the channel was activated and auditory perception elicited. Without auditory perception but an active channel, a yellow color is used. A deactivated electrode contact is colored in grey. The black hashed contact indicates channel E09 of subject A-01 with high impedance. This electrode contact was deactivated with no measurements being done. The green hashed contact indicates channel E05 of subject A-15. Although an auditory perception could be elicited, it had been deactivated due to a lack of loudness growth. In this case, a behavioral THR level could be estimated, but no MCL level. If a LEP response could be observed, the channel is marked by a red circle, otherwise by a black circle. An AGF slope of less than 10 μV/qu is regarded as shallow and indicated by a dotted red circle.

### Correlation between objective LEP threshold and behavioral fitting parameters

When channels with auditory perception are activated, at least minimum auditory performance should be achieved, characterized by a CAP value above zero. Although this expectation was met, there were two exceptions. Patient A-14 did not use his ABI since only one channel (E12) delivered an auditory perception. In child A-05, both auditory perception and LEP responses were found only at the initial fitting (see Fig 4). At that time, estimation of fitting parameters was preliminary. The follow-up visit 40 days later showed no auditory perception and no LEP response, and fitting parameters could not be determined. An electrode migration was suspected, but was not confirmed by imaging so far. The patient came from abroad and did not show up for further appointments.

**Fig 5:**
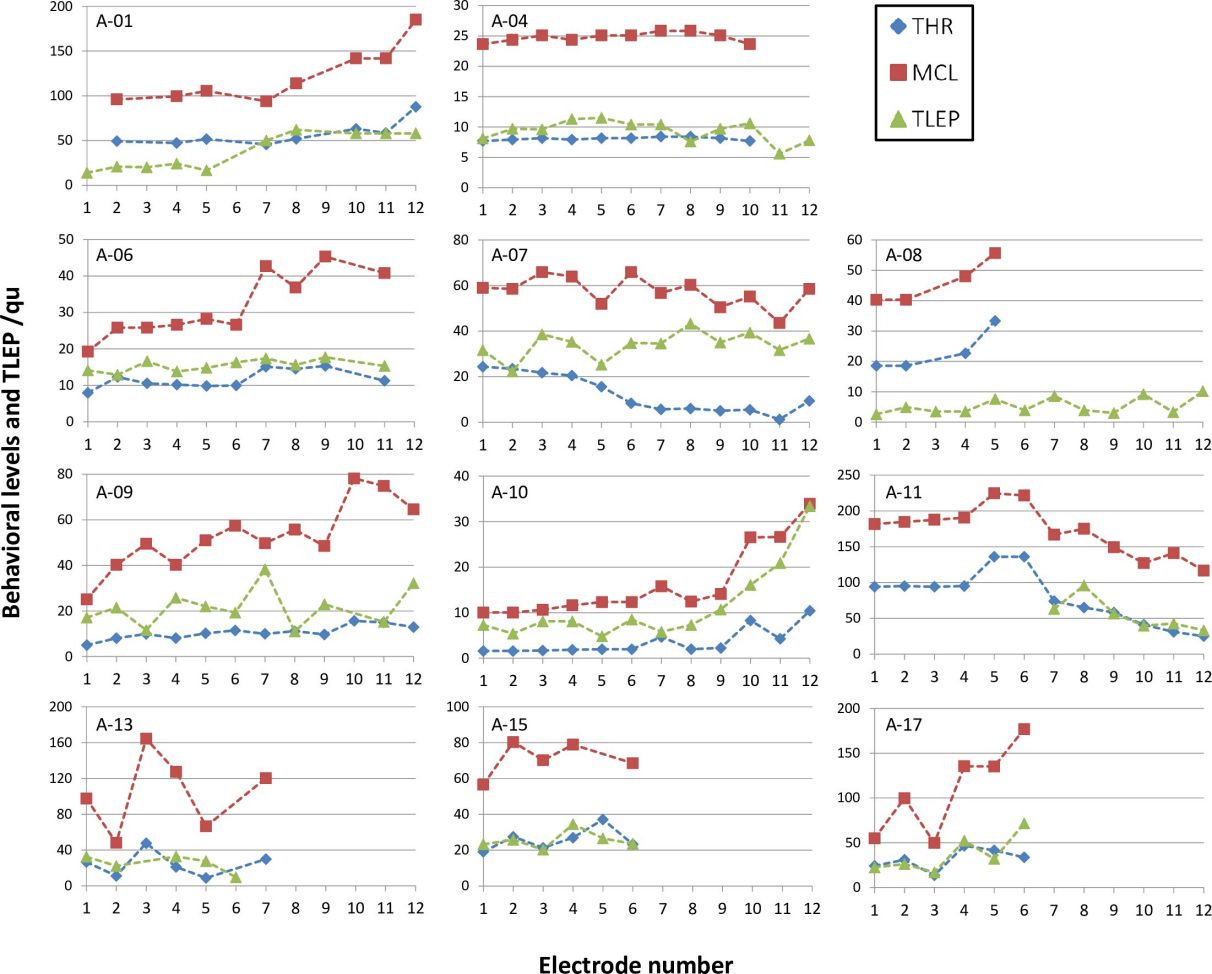
Objective and behavioral levels. Behavioral parameters THR and MCL and objective thresholds TLEP are shown for patients with auditory performance (CAP > 0).

**Fig 6:**
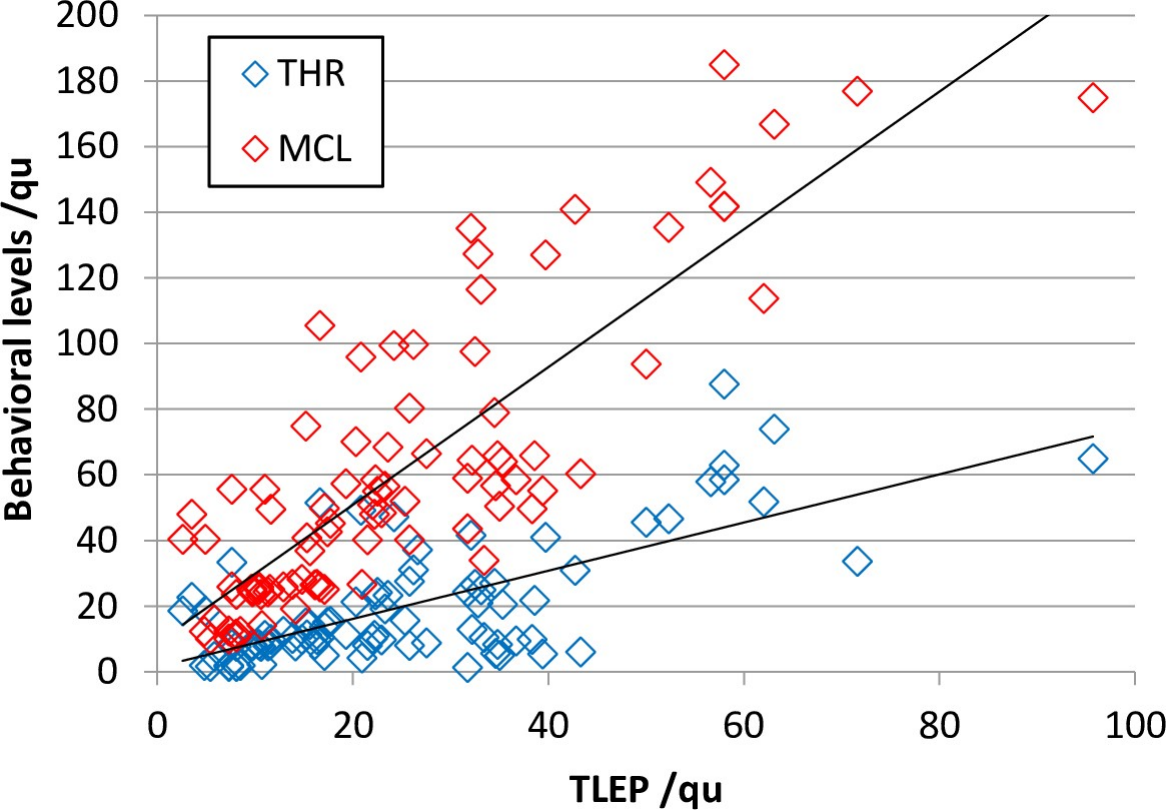
Correlation of objective and behavioral levels. From 11 patients with auditory performance (CAP > 0) in total 89 THR and 88 MCL levels were available for comparison with TLEP data.

In eleven subjects, CAP values between 1 and 4 were achieved so far, while five subjects did not benefit from the ABI at all (see Table 1). From the first subgroup, the behavioral fitting parameters THR and MCL as well as the objective TLEP are shown in Fig 5 for the whole electrode array. Fig 6 shows the correlation between behavioral parameters and objective thresholds. We found a correlation between TLEP and THR with a correlation coefficient (Pearson) of r = 0.697 and between TLEP and MCL with r = 0.840. Both correlations were highly significant (p < 0.001).

## Discussion

In [14] we described the LEP measurement method referring to child A-04. In the current retrospective study, LEP recordings of a larger cohort of ABI patients were analyzed. To our knowledge, this is the first study, reporting clear LEP responses, which can be compared to ECAP responses recorded in CI patients.

The focus of this retrospective study was on the usability of LEP measurements recorded during the clinical routine, rather than analyzing response patterns in detail. Even without parameter optimization, LEP measurements predicted auditory perception in ABI users with a sensitivity of 90.5%.

### Neuronal population response

The neuronal source of ECAP responses is very well researched and documented. EABR measurements in rats have shown that the first positive wave (P1), the compound action potential of the auditory nerve, and also, but to lesser degree, the N1-P2 complex correlates with the number of surviving spiral ganglion neurons [15]. This N1-P2 complex can be recorded by ECAP measurements, too.

However, with an ABI electrode, multiple subtypes of neurons in the cochlear nucleus are likely to be stimulated [16]. Therefore, a LEP response is not necessarily connected to auditory perception, which may explain the false positive rate of 33.7%. When neurons attributed to auditory perception, are responding together with non-auditory neurons, LEP threshold may be shifted towards lower values. Indeed, TLEP was found to be even lower than THR on several electrode contacts (Fig 5). However, more reasons for such low TLEP values can be identified. In CI users, objective ECAP thresholds are usually well above behavioral thresholds. The distance between the auditory nerve and the intra-cochlear electrodes is larger than between the sub-dural ABI electrodes and the cochlear nucleus. Furthermore, in CIs there is bone tissue between the neural tissue and the intra-cochlear electrodes. This would explain the need for a stronger stimulus in CI to evoke an action potential in comparison to ABI. In addition, stimulation rate has an influence on determining levels [17]. LEP measurements were carried out at approximately 76 pps, whereas behavioral thresholds were estimated with pulse bursts of few hundreds pps. The uncertainty of assessing behavioral levels, especially in young children, will add another possible explanation. At least in the adult subgroup (A-13, A-15, and A-17), TLEP and THR were at about the same level. As children grow older, more reliable assessments of behavioral levels will be possible.

P peaks were found at different latencies. The shortest, at around 400 μs, matches the intrinsic time constant of chopper neurons, which are the main projecting neurons to the ascending auditory system [18]. Detailed analysis of latencies and raw data will be examined in a future study to address the question whether LEP measurements are suitable for differentiating auditory from non-auditory perception. In this study, only TLEP as a value that can be retrieved directly from the clinical software, and which can be integrated directly into the fitting map, was considered. Recently, Azadpour et al. [19] have shown that assessing the temporal responsiveness by extended LEP measurements (recovery function) may provide further information about responding neurons in the cochlear nucleus. Measuring channel interaction by means of another extended LEP measurement (spread of excitation) would offer further insight into excitability of the neuronal population at the cochlear nucleus. This could lead to improved speech coding strategies, specifically for ABI patients. However, such extended measurements need much more time and are currently not part of our clinical routine.

LEP responses may be strong enough to make artifact reduction unnecessary. In S1 Fig, an example is given showing raw responses underlying the curves in Fig 2. Both anodic and cathodic leading parts are shown prior to the application of zero amplitude template and rectification. In this case, P peak latency was lower for the cathodic leading phase. S2 Fig equally shows raw responses underlying the records presented in Fig 3. No P peak can be observed here.

Especially in congenitally deaf children, where the development of the auditory system and language commences only after ABI implantation, electrode contacts are checked regularly for auditory perception. It may happen that a formerly deactivated electrode will be activated or vice versa. It would be interesting to know if LEP responses would also reflect such a change in electrode status. Unfortunately, our limited data cannot answer this question for now.

### False negative: No LEP response despite auditory perception

**Fig 7:**
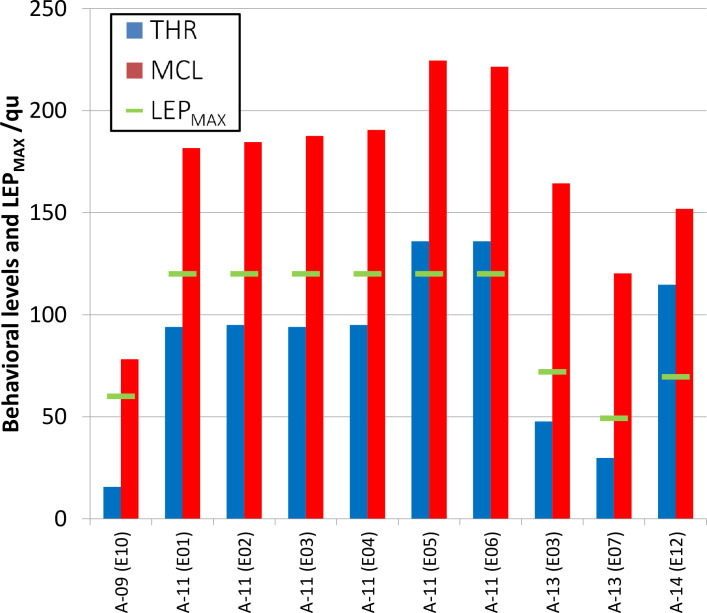
LEP measurements with a false negative result regarding auditory perception. On 10 electrode contacts, a TLEP could not be estimated although an auditory perception was elicited. The maximum stimulus charge LEP_MAX_, available or chosen for measurement, is shown in comparison to the behavioral parameters THR and MCL.

Although no LEP response was observable, an auditory perception could be elicited on 10 electrode contacts, corresponding to a false negative rate of 9.5%. We analyzed these cases regarding behavioral parameters THR and MCL and the maximum stimulus charge LEP_MAX_ prevailing during the measurement (Fig 7).

LEP_MAX_ will never exceed 120 qu, since this limit is set by the manufacturer. However, LEP_MAX_ may be well below 120 qu due to three different reasons: Firstly, any finite impedance value will have an impact on the compliance limit, above which the stimulus charge will saturate. Secondly, if a response exceeds +/- 5 mV, the implant amplifier may be saturated or the output gets clipped. The clinical software will indicate when such limits are reached and affected recordings have been excluded from our analysis. Thirdly, measurements were conducted during the clinical routine and different upper limits were set manually, e.g. by choosing PD being below maximum. In addition, when side effects or any uncomfortable sensations occurred, the measurement was aborted. Details about LEP_MAX_ are listed in S1 Table.

The determination of a threshold by a regression line requires several measurement points at different stimulus levels above this threshold. For that reason, TLEP is supposed to be well below LEP_MAX_. If behavioral THR is close to or exceeds LEP_MAX_, it is not surprising that a LEP response is not observable despite auditory perception. Taking this into account, the sensitivity of LEP measurements would be even higher.

The choice of PD is expected to have an impact on the recorded responses. If PD is too short, stimulus charge will be low, and a TLEP may not be found. If PD is too long, clipping or saturation may occur. In addition, the recording electrode may have an influence. If the recording electrode is more distant to the stimulating electrode, the recorded response will be smaller and clipping or saturation of the amplifier is less likely. Further optimization of measurement parameters like PD is necessary to improve the sensitivity of the LEP method.

### Benefit of LEP for fitting

Fitting auditory prostheses in young children is challenging since those patients cannot give reliable feedback upon the quality of their sensation evoked by electrical stimulation. Non-auditory side effects occurring in CI are mainly confined to facial nerve stimulation and visually detectable by the audiologist. In ABI patients, additional side effects like coughing and tingling may occur. These subtle sensations may be hard to observe. For that reason, objective measures, which can distinguish auditory from non-auditory sensations, would be desirable. We were unable to correlate side effects with of LEP measurement results, because stimulation was stopped immediately when the patient complained about unpleasant sensations. So far, we would not recommend to conduct fitting based on TLEP alone. Future studies and further analysis are necessary to determine reliable predictors for auditory perception. Especially in congenitally deaf children, developmental delay may compromise behavioral reactions on electrical stimuli, impeding on speech processor programming. LEP measurements may indicate promising electrode contacts which should then be kept activated in favor of those channels lacking LEP responses. With Cis, the electrode array will be inserted into the cochlea and will stay there relatively fixed. Although electrode migrations may happen [20,21], this issue is manageable. At our clinic, improved surgery techniques were successfully implemented to prevent migrations. However, fixing an ABI electrode array in the lateral recess is much more challenging. Here, migration may occur more often compared with CIs. Furthermore, a possibly migration of the ABI electrode array is subject to three degrees of freedom, since it could be shifted into or away from the ventricle or be rotated in itself. Regardless of the type of neurons responding to an electric stimulus, response patterns should change with migration, which could be utilized to detect or at least suspect any such migration. Ultimately, this will be proved by imaging (digital volume tomography). During surgery, EABR measurements usually support the placing of the ABI electrode array on the cochlear nucleus. A high number of trials (typically 1000) is needed to reduce background noise by signal averaging. Furthermore, these far-field recordings are susceptible to environmental distortions. When the final position of the array is found, LEP measurements could serve recording baseline patterns. Since fewer trials (typically 30) are necessary to record such local-field responses, shorter measurement duration is required. Continuous LEP measurements could help monitoring array position during the final part of surgery and wound closure. Measurements can be repeated any time postoperatively to check for proper electrode position as a prerequisite for fitting.

Future studies should incorporate correlation analysis of EABR thresholds with TLEPs. However, EABR measurements would have to be carried out in anesthesia, at least under sedation, because any patients’ movement will cause an artifact compromising response. Monitoring LEP during ABI implantation surgery would offer an opportunity for those comparisons.

## Conclusions

LEP can be measured without sedation using the same equipment needed to conduct the fitting. In our group of ABI patients, implanted with a device by MED-EL, it was possible to record responses from the cochlear nucleus with the implanted ABI electrode array. LEP thresholds correlate well with behavioral parameters THR and MCL. Therefore, LEP measurements have the potential to support the fitting in ABI patients, although they have not been proved to be a reliable predictor to distinguish auditory from non-auditory perception yet. Since LEP responses can be attributed to the activity of evoked neurons in the vicinity of the electrode contacts, the position of the whole electrode array should be verifiable without the burden of radiation through imaging. Therefore, measuring LEP together with EABR intraoperatively is recommended. For the time being, LEP measurements do not relieve of the elaborate work of behavioral fitting yet.

## Supporting information

S1 Fig Raw responses underlying Fig 2. Raw responses from A. anodic, and B. cathodic leading parts before AP, zero amplitude template and rectification were applied. Subject A-10.
(TIF)

S2 Fig Raw responses underlying Fig 3. Raw responses from A. anodic, and B. cathodic leading parts before AP, zero amplitude template and rectification were applied. Subject A-02.
(TIF)

S1 Table Data from objective LEP measures and behavioral fitting. (XLSX)
