# Supplementary figures and images for: Measurements of the local evoked potential from the cochlear nucleus in patients with an auditory brainstem implant and its implication to auditory perception and audio processor programming
Source: PLoS One. 2021 Apr 1;16(4):e0249535. doi: 10.1371/journal.pone.0249535 (PMC8016231; doi:10.1371/journal.pone.0249535)

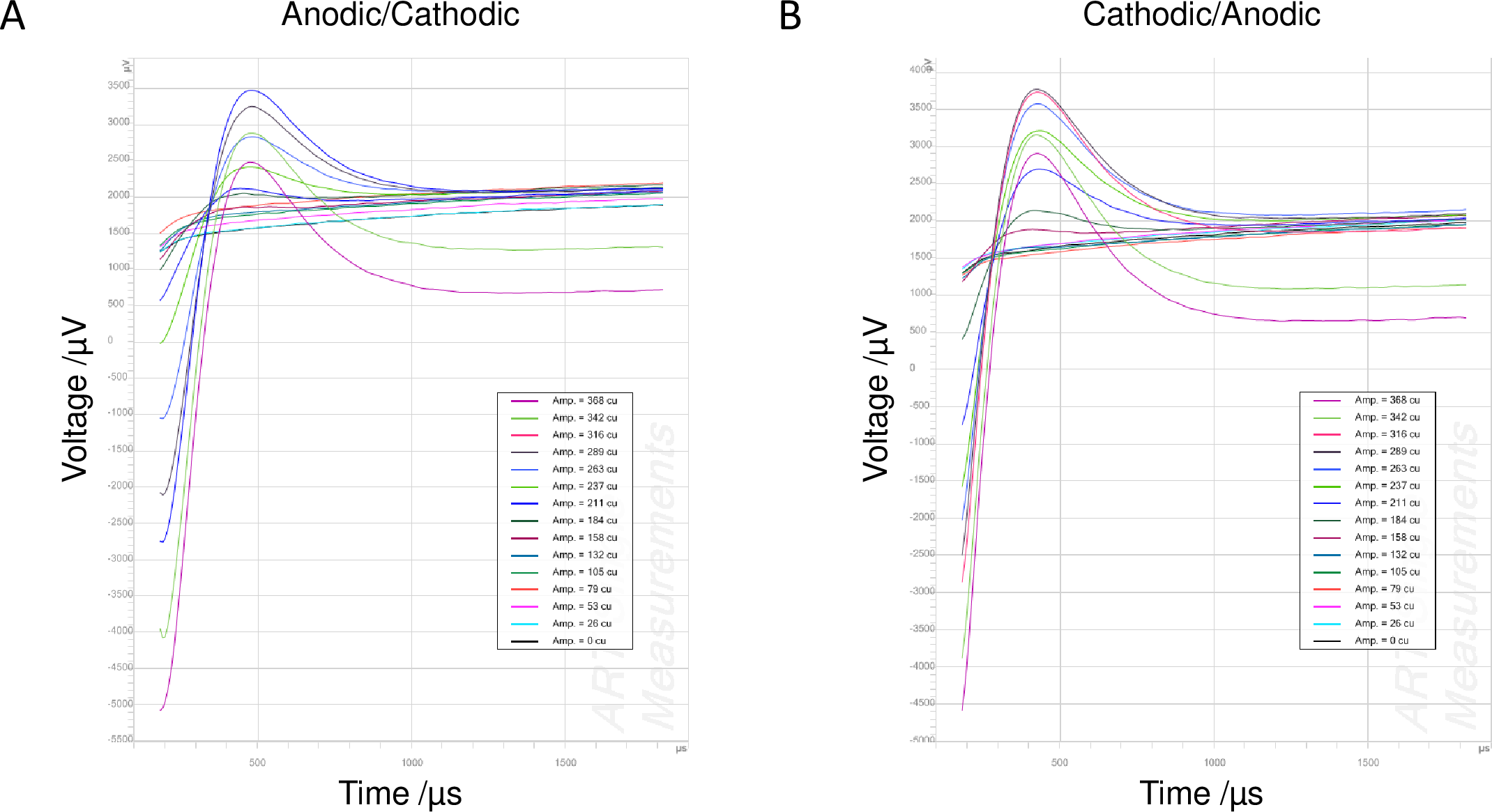

Supplement: S1 Fig — Raw responses from A. anodic, and B. cathodic leading parts before AP, zero amplitude template and rectification were applied. Subject A-10. (TIF) [file pone.0249535.s001.tif]

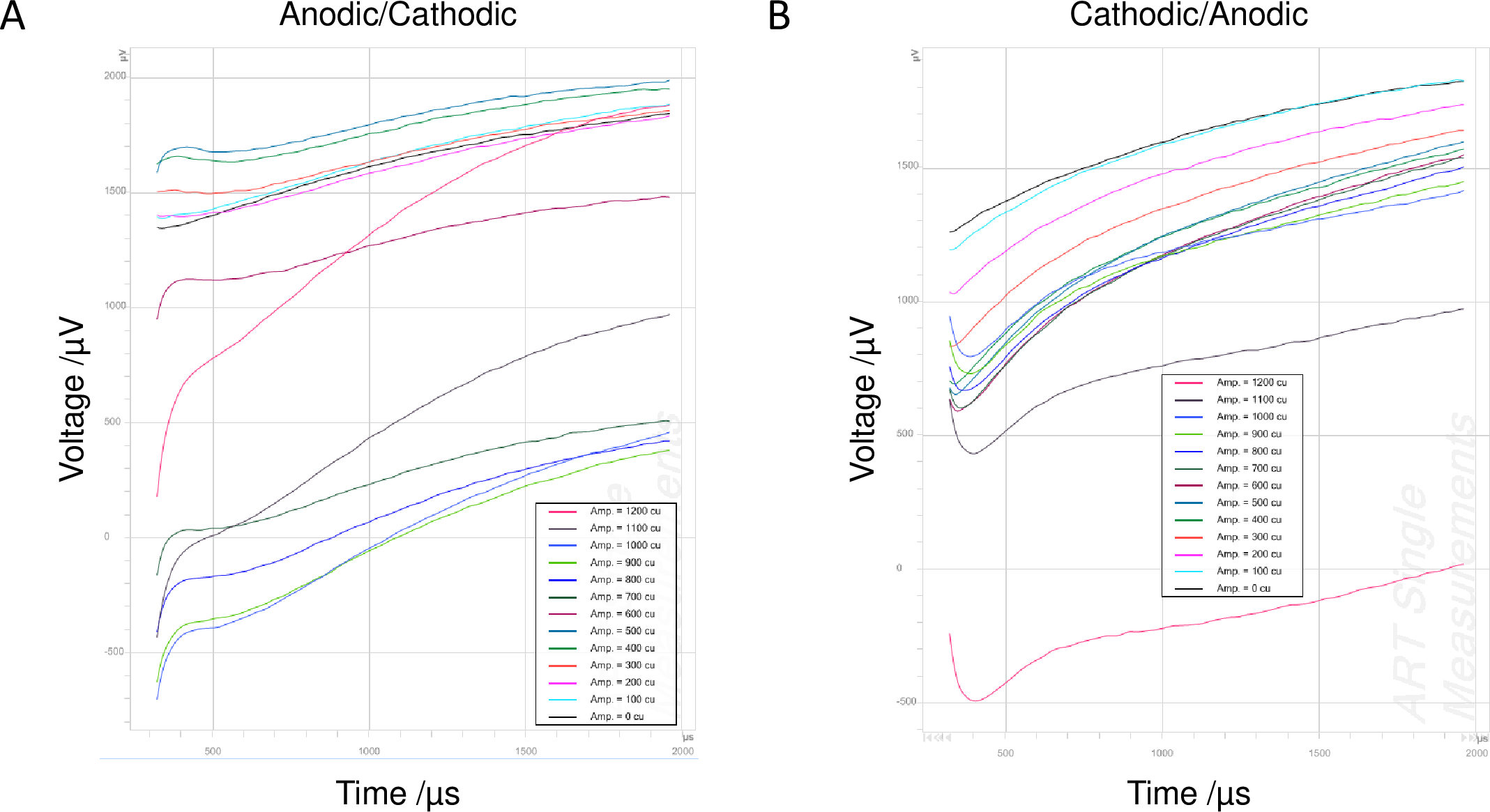

Supplement: S2 Fig — Raw responses from A. anodic, and B. cathodic leading parts before AP, zero amplitude template and rectification were applied. Subject A-02. (TIF) [file pone.0249535.s002.tif]
